# The influence of institutional pancreaticoduodenectomy volume on short-term outcomes in the Brazilian public health system: 2008-2021

**DOI:** 10.1590/0100-6991e-20233569-en

**Published:** 2023-08-14

**Authors:** DANIEL JOSÉ SZOR, FRANCISCO TUSTUMI

**Affiliations:** 1 - Hospital Israelita Albert Einstein, Ciências em Saúde - São Paulo - SP - Brasil

**Keywords:** Pancreaticoduodenectomy, Pancreatic Neoplasms, Pancreatic Diseases, Hospital Mortality, Delivery of Health Care, Quality Improvement, Pancreaticoduodenectomia, Neoplasias Pancreáticas, Pancreatopatias, Mortalidade Hospitalar, Prestação Integrada de Cuidados de Saúde, Melhoria de Qualidade

## Abstract

**Introduction:**

pancreaticoduodenectomy is a complex surgical procedure that can result in high rates of complications and morbimortality. Due to its complexity, the establishment of referral centers has increased in recent decades. This study aims to evaluate the influence of the institutional volume of pancreaticoduodenectomy for periampullary cancer on short-term outcomes in the Brazilian public health system.

**Methods:**

this study used a population-based approach and investigated the number of pancreaticoduodenectomies performed by institutions within Brazil’s public health system between 2008 and 2021. High-volume institutions were defined as those that performed more than two standard deviations above the mean number of procedures per year. Specifically, if a center performed eight or more pancreaticoduodenectomies annually, it was considered a high-volume institution.

**Results:**

in Brazil, 283 public hospitals performed pancreaticoduodenectomy for cancer between 2008 and 2021. Only ten hospitals performed at least eight pancreaticoduodenectomies per year, accounting for approximately 3.5% of the institutions. High-volume institutions had a significantly lower in-hospital mortality rate than low-volume institutions (8 vs. 17%). No significant differences between groups were observed for length of stay, hospitalizations using the ICU, and ICU length of stay. The linear regression model showed that the number of hospital admissions for pancreaticoduodenectomy and age were significantly associated with hospital mortality.

**Conclusion:**

institutional pancreaticoduodenectomy volume implies a lowering of in-hospital mortality. The findings of this nationwide study can affect how the public health system manages pancreaticoduodenectomy care.

## RESUMO

**Introdução:**

a duodenopancreatectomia é um procedimento complexo com alta risco de complicações e morbimortalidade. Devido à sua complexidade, a constituição de centros de referência tem aumentado nas últimas décadas. Este estudo tem como objetivo avaliar a influência do volume institucional de duodenopancreatectomia para câncer periampular em resultados de curto prazo no sistema público de saúde brasileiro.

**Métodos:**

este estudo utilizou uma abordagem de base populacional e investigou o número de duodenopancreatectomia realizadas por instituições do sistema público de saúde do Brasil entre 2008 e 2021. Definimos instituições de alto volume como aquelas que realizaram mais de dois desvios padrão acima do número médio de procedimentos por ano. Especificamente, um centro seria considerado de alto volume se realizasse oito ou mais duodenopancreatectomias anualmente.

**Resultados:**

no Brasil, 283 hospitais públicos realizaram duodenopancreatectomia por câncer entre 2008-2021. Apenas dez hospitais realizaram pelo menos oito duodenopancreatectomias anualmente, correspondendo a aproximadamente 3,5% das instituições. As instituições de alto volume tiveram uma taxa de mortalidade intra-hospitalar significativamente menor do que as instituições de baixo volume (8 vs. 17%). Não foram observadas diferenças significativas entre os grupos quanto ao tempo de internação, internações na UTI e tempo de internação na UTI. O modelo de regressão linear mostrou que o número de internações para duodenopancreatectomia e a idade estiveram significativamente associados à mortalidade hospitalar.

**Conclusão:**

o volume institucional da duodenopancreatectomia implica em diminuição da mortalidade intra-hospitalar. Os achados deste estudo nacional podem afetar a forma como o sistema público de saúde gerencia os cuidados com a duodenopancreatectomia..

## INTRODUCTION

Pancreaticoduodenectomy (PD) is a complex surgical procedure that can result in high rates of complications and morbimortality. The majority of patients who are candidates for this procedure have an oncological diagnosis, which is an additional challenge for patients prognosis^1^.

Due to its complexity, the establishment of referral centers for its performance has been spreading in recent decades. The centralization of complex surgical procedures into a few specialized institutions has been proposed to improve results through the standardization of surgical technique and postoperative care^2^ ^-^ ^5^. The definition of a high-volume center for PD is not established universally. Studies indicate different cutoffs, ranging from 2 to 125 PD annually, to characterize a high-volume center^6^ ^-^ ^10^.

In Brazil, most surgical procedures are carried out by the national public health system (SUS). Currently, there is no consensus on how to manage periampullary tumors in a large public system such as SUS. SUS guarantees universal access, meaning that everyone in the national territory is covered by this public healthcare system-making it the world’s largest public health system worldwide^11^ ^,^ ^12^.

This study aims to evaluate the influence of the institutional volume of PD for periampullary cancer on short-term outcomes in the Brazilian public health system. 

## MATERIALS AND METHODS

This study, which employed a population-based approach, encompassed the inclusion of both available codes on the platform to include all cases submitted to duodenopancreatectomy through the Brazilian Public Health System (SUS). The data utilized in the analysis were extracted from DATASUS, the informatics branch of Brazil’s public health system. Specifically, we relied on the SUS Procedures, Medicines, and OPM Table Management System (SIGTAP) codes ‘04.16.04.012-8’ for ‘duodeno pancreatectomy’ and ‘04.07.03.020-4’ for ‘pancreato duodenectomy’.

However, it is important to acknowledge that this study has a limitation due to the reliance on manual inclusion of cases in the platform, which can result in underreporting or sub-notification of surgeries. The potential for incomplete data entry poses a constraint on the study’s findings and must be taken into consideration when interpreting the results.

The data on the number of hospitalizations per patient, as identified by the Hospitalization Authorization (AIH), were obtained from TabNet/DATASUS. Information on the length of stay (LOS), ICU stay, number of in-hospital deaths, and hospital costs for each hospitalization was also extracted. The population sizes for each Brazilian federative unit were calculated using data from the Brazilian Institute of Geography and Statistics (IBGE) from 2015.

The number of hospitalizations related to PD for each Brazilian federative unit was expressed as a rate per 100,000 inhabitants using population size. The incidence graph was created using TabNet/DATASUS and Microsoft 365 (Office) Excel spreadsheet software.

High-volume and low-volume institutions were compared. High-volume institutions were defined as those that performed an average of at least eight PDs per year from 2008 to 2022. Student’s t-test was used to determine differences between groups for continuous variables, considering unequal variances. A 0.05 significance level was adopted. Linear correlation analysis was used to examine the associations between surgical volume and length of hospital stay, ICU stay, the proportion of patients demanding postoperative ICU care, and in-hospital mortality and was expressed as coefficient and robust standard errors (SE). Covariates with a p-value <0.05 in univariate analysis were selected for multivariate analysis. Scatter plots with the corresponding fit lines are also presented.

We chose a cutoff of 8 annual PD based on evaluating which institution performed more than two standard deviations above the mean number of procedures per year, corresponding to Brazil’s top 3.5% volume hospitals.

Statistical analyses were performed with STATA 16.1 Software (StataCorp 4905 Lakeway Drive College Station, Texas 7).

The study was approved by the local Ethics Committee (SGPP 5338-22) and did not require informed consent forms to be obtained. The raw data used in the study are publicly accessible. The STROBE statements were followed^13^.

## RESULTS

**Figure 1:**
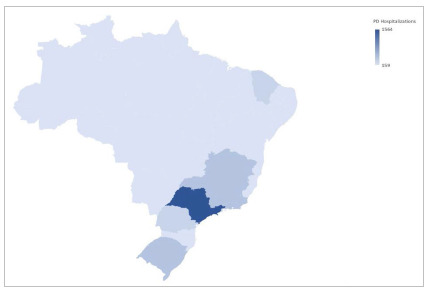
Number of hospitalizations for pancreaticoduodenectomy according to the Brazilian regions. PD: Pancreaticoduodenectomy.

**Figure 2:**
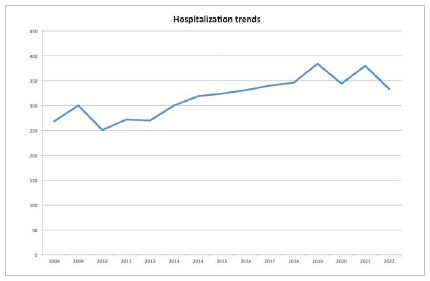
Hospitalization for pancreaticoduodenectomy in Brazil over the years.

**Figure 3:**
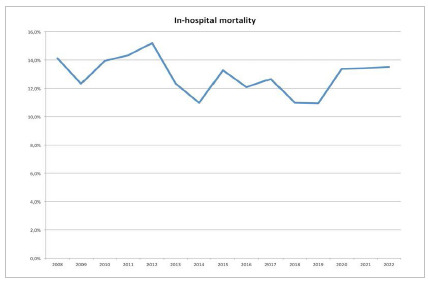
In-hospital mortality after pancreaticoduodenectomy in Brazil over the years.

In Brazil, 283 public hospitals performed 4,763 PDs for cancer between 2008 and 2021. Most procedures were performed in the Brazilian Southeast region, which accounted for 57% of total procedures (Figure 1). There was a trend in increasing the annual number of hospitalizations for PD in the country, but the in-hospital mortality was similar over the years (Figures 2 and 3).

Pancreatic cancer was the most common diagnosis (51.3%), followed by cholangiocarcinoma (21.7%) and duodenal cancer (18.9%). 

Only ten hospitals performed at least 8 PDs per year (high-volume institutions), accounting for approximately 3.5% of the institutions. Between 2008 and 2021, high-volume institutions had a mean number of hospitalizations of 156.7 (41.77), while low-volume institutions had a mean of 11.71 (16.68). High-volume institutions had a significantly lower in-hospital mortality rate than low-volume institutions (8 vs. 17%). No significant differences between groups were observed for LOS, hospitalizations using the ICU, and ICU length of stay. The mean age of patients treated in high-volume institutions was slightly higher than those treated in low-volume institutions (59.32 vs. 57.74 years old). Table 1 summarizes the comparisons between high- and low-volume institutions.

**Table 1:** Comparisons between high- and low-volume institutions. LOS: Length of stay; ICU: Intensive care unit; SD: Standard deviation. Significant p-values were demonstrated with*.

	High-volume		Low-volume		
n	10		273		
	Mean	SD	Mean	SD	p-Value
Hospitalizations	156.70	41.77	11.71	16.68	<0.001*
In-hospital mortality	0.08	0.06	0.17	0.25	0.001*
LOS	15.46	5.36	14.39	9.31	0.561
% Hospitalizations using ICU	70.31	22.43	70.92	32.60	0.936
ICU length of stay	3.29	1.64	3.48	3.39	0.744
Age	59.32	1.28	57.74	8.51	0.019*

**Figure 4:**
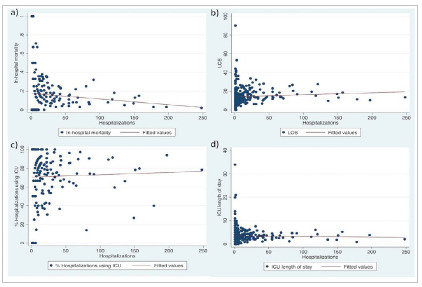
Scatter plot evaluating the number of hospitalizations for pancreaticoduodenectomy and the outcomes a) In-hospital mortality; b) Length of hospital stay; c) percent of patients demanding ICU care; and d) ICU length of stay.

In univariate analysis, the linear regression model showed that the number of hospital admissions for PD was inversely associated with hospital mortality (Coef: -10.667; p=0.009). In-hospital mortality and patient age were selected for multivariate analysis, and both were considered significant in the final model (Table 2 and Figure 4).

**Table 2:** Linear regression evaluating the outcome in-hospital mortality. PD: pancreatoduodenectomy; LOS: length of stay; ICU: intensive care unit; Coef: coefficient; SE (Robust): robust standard error; CI: confidence interval; LL: lower limit; UL: upper limit. Significant p-values were demonstrated with *.

	Univariate					Multivariate				
	Coef.	SE (Robust)	p-Value	95% CI		Coef.	SE (Robust)	p-Value	95% CI	
				LL	UL				LL	UL
Hospitalizations for PD	-10.667	4.084	0.009*	-18.707	-2.628	-11.320	4.181	0.007*	-19.550	-3.089
LOS	0.251	0.172	0.147	-0.088	0.591					
% Hospitalizations using ICU	0.0252	0.044	0.566	-0.061	0.111					
ICU length of stay	-0.243	0.318	0.446	-0.868	0.387					
Age	0.261	0.100	0.010*	0.064	0.458	0.283	0.104	0.007*	0.078	0.488

## DISCUSSION

This large public health system study showed that institutional experience with PD impacts postoperative outcomes. This study’s findings might impact the management of administrative capacities, resources, and organizational structures for optimizing the treatment of periampullary cancers.

PD is a technically demanding surgery that requires specialized skills from the surgeon. The critical location of periampullary neoplasms means that cancer resection comprises gastric, biliary, duodenal, and potentially vascular resections and reconstructions. Accordingly, periampullary neoplasm management demands the institutional availability of high-quality hepatobiliary and vascular surgeons, oncologists, endoscopists, and intensivists, among others. Each of these specialties must overcome a particular learning curve to achieve excellence. The institutional surgical volume translates the sum of the experience and learning acquired over the years of each specialty within the institution.

Müller et al.^14^, in a meta-analysis, found that the minimum number of PDs for a surgeon to surpass the first phase of the learning curve is 39 for laparoscopic, 30 for open, and 25 for robotic surgeries. However, definitions of learning curves are heterogeneous among studies. Depending on the definition, a minimum of 7 to 250 cases have been described thus far for achieving proficiency in PD (15-22).

The cutoff value to define a high-volume center for PD has yet to be defined. Identifying a minimal number that is associated with reduced mortality and complications is a challenging task. We chose a cutoff of 8 annual PD based on evaluating which institution performed more than two standard deviations above the mean number of procedures per year, corresponding to Brazil’s top 3.5% volume hospitals. However, many experts would agree that eight annual PD might be a low volume for such a complex procedure. Panni et al.^23^ recommend defining low-volume hospitals as those performing 1-9 pancreaticoduodenectomies per year, medium-volume hospitals as those performing 10-36, and high-volume hospitals as those performing over 36 pancreaticoduodenectomies per year. The PD is dissolved among several institutions that perform PD in Brazil. Due to the lack of control, numerous institutions with no expertise in surgical oncology and complex procedures perform PD. 

The Brazilian healthcare system probably lacks more significant control and centralization of PD care, which is a highly complex procedure and demands institutional expertise and expensive resources. Despite SUS stratifying healthcare into four levels of complexities, in which the most complex conditions would be treated in the third and fourth levels, maybe highly complex procedures, including PD, should be centered in only a few specialized institutions, following the strategy previously determined by the Brazilian healthcare system for liver transplantation services^24^. The third and fourth levels of complexity hospitals are usually overwhelmed, with high occupancy rates, and complex conditions end up being managed in hospitals with no expertise in oncology. Botega et al.^25^ analyzed the spatial organization of the Brazilian health system and highlighted the need to reallocate resources to potentialize hospital utilization of inpatient care without increasing access inequities.

Concentrating PD in centers of excellence is an attractive solution for managers, improving postoperative outcomes and potentially reducing treatment-related costs. This strategy might be even more relevant in large public health systems, where demands for complex procedures are high while resources are scant. This strategy is also suggested for other conditions that demand complex care. Anacleto et al.^26^ evaluated thoracoabdominal aortic aneurysm repair in the Brazilian Public Health System and suggested the creation of specialized referral centers. 

Our data show that PD in-hospital mortality in Brazilian centers decreases from 16% to 8% when the patient is managed in centers of excellence. Due to the population-based nature of this study, some data regarding the cause of mortality and surgical complications are lacking. Although our findings do not demonstrate differences in LOS and ICU stay, the DATASUS database sums the length of stay for every patient, including those who die during hospitalization. Namely, in this database, patients who developed severe complications and eventually died were not excluded from the analysis. This may be misrepresented as a short stay in low-volume institutions, which had higher mortality.

The mean age of patients in high-volume institutions was significantly higher than that of patients in low-volume institutions. Referral centers probably had lower thresholds for indicating surgery, while institutions with low expertise in PD were more selective and indicated surgery for younger patients and fitter for surgery. After multivariate analysis, institutional volume and patients’ mean age were covariates significantly associated with in-hospital mortality.

Previous publications also investigated the institutional PD volume. Balzano et al.^27^ evaluated 12,662 surgeries performed in 395 hospitals in Italy and showed that hospital mortality increased from 3.1% in high-volume centers to 10.6% in low-volume centers. Panni et al.^23^, analyzing data from the National Cancer Database, selected 42,402 patients who underwent PD and found that hospitals with more than nine cases per year had improvement in 90-day mortality. Our study evaluated elective PD performed in the Brazilian Unified Health System (SUS) with similar results. SUS is a public healthcare system in Brazil that aims to ensure universal, integral, and equitable access to health services for all citizens, as well as promote health and prevent diseases. SUS includes a network of public hospitals, primary care centers, specialized clinics, and disease prevention and health promotion programs. We obtained all data from DATASUS, an open-access database that provides information on various health indicators in SUS. DATASUS is used to monitor and evaluate the performance of the SUS system, identify trends and disparities in the provision of health services, and develop targeted interventions to improve the population’s overall health.

This population-based study has some limitations. First, it covered only the Brazilian public health system, and consequently, private institutions were not studied. The private system may have different postoperative outcomes, due to difference in funding and resources availability. Besides, population-based studies are prone to information bias due to sub-notification. In addition, there must be an imponderable limitation on the validity of applying the findings of the current study outside the setting of Brazilian public health. The profile of the periampullary tumors in other countries may vary slightly. For example, Kamarajah et al.^28^, based on the North American database Surveillance, End Results and Epidemiology (SEER), studied 9,877 patients who underwent surgery between 2005 and 2013 and found that 79% had pancreatic ductal adenocarcinoma, 6% had distal cholangiocarcinoma, 4% had duodenal carcinoma, and 11% had ampullary carcinoma. Our study presented slightly different proportions for each PD diagnosis.

Knowledge based on data analysis from SUS, the world’s largest public health system, might impact how health managers centralize periampullary neoplasm care. Future ecological studies from different parts of the world are still needed to check external validity and detect covariables that may influence postoperative outcomes.

## CONCLUSION

Institutional high PD volume implies lower in-hospital mortality. The findings of this nationwide study can affect how public health manages PD care and its resources.
